# Neighborhood walk score and selected Cardiometabolic factors in the French RECORD cohort study

**DOI:** 10.1186/s12889-017-4962-8

**Published:** 2017-12-19

**Authors:** Julie Méline, Basile Chaix, Bruno Pannier, Gbenga Ogedegbe, Leonardo Trasande, Jessica Athens, Dustin T. Duncan

**Affiliations:** 1 Sorbonne Universités, UPMC Univ Paris 06, UMR_S 1136, Pierre Louis Institute of Epidemiology and Public Health, 1 rue Victor Cousin, 75230, 05 Paris cedex, France; 2 Inserm, UMR_S 1136, Pierre Louis Institute of Epidemiology and Public Health, 56, boulevard Vincent Auriol CS 81393, 75646 Paris Cedex 13, France; 3 IPC Medical Center, 6 Rue la Pérouse, 75016 Paris, France; 4 Department of Population Health, New York University School of Medicine, 227 East 30th Street, New York, NY 10016 USA; 5 Departments of Pediatrics and Environmental Medicine, New York University School of Medicine, 550 First Avenue, New York, NY 10016 USA; 6 Spatial Epidemiology Lab, Department of Population Health, New York University School of Medicine, 227 East 30th Street, 6th Floor, Room 621, New York, NY 10016 USA

**Keywords:** Built environment, Walkability, Walk score, Cardiovascular disease, Paris

## Abstract

**Background:**

Walkable neighborhoods are purported to impact a range of cardiometabolic outcomes through increased walking, but there is limited research that examines multiple cardiometabolic outcomes. Additionally, few Walk Score (a novel measure of neighborhood walkability) studies have been conducted in a European context. We evaluated associations between neighborhood Walk Score and selected cardiometabolic outcomes, including obesity, hypertension and heart rate, among adults in the Paris metropolitan area.

**Methods and results:**

We used data from the second wave of the RECORD Study on 5993 participants recruited in 2011–2014, aged 34–84 years, and residing in Paris (France). To this existing dataset, we added Walk Score values for participants’ residential address. We used multilevel linear models for the continuous outcomes and modified Poisson models were used for our categorical outcomes to estimate associations between the neighborhood Walk Score (both as a continuous and categorical variable) (0–100 score) and body mass index (BMI) (weight/height^2^ in kg/m^2^), obesity (kg/m^2^), waist circumference (cm), systolic blood pressure (SBP) (mmHg), diastolic blood pressure (DBP) (mmHg), hypertension (mmHg), resting heart rate (RHR) (beats per minute), and neighborhood recreational walking (minutes per week). Most participants lived in Walker’s Paradise (48.3%). In multivariate models (adjusted for individual variables, neighborhood variables, and risk factors for cardiometabolic outcomes), we found that neighborhood Walk Score was associated with decreased BMI (β: -0.010, 95% CI: -0.019 to −0.002 per unit increase), decreased waist circumference (β: -0.031, 95% CI: -0.054 to −0.008), increased neighborhood recreational walking (β: +0.73, 95% CI: +0.37 to +1.10), decreased SBP (β: -0.030, 95% CI: -0.063 to −0.0004), decreased DBP (β: -0.028, 95% CI: -0.047 to −0.008), and decreased resting heart rate (β: -0.026 95% CI: -0.046 to −0.005).

**Conclusions:**

In this large population-based study, we found that, even in a European context, living in a highly walkable neighborhood was associated with improved cardiometabolic health. Designing walkable neighborhoods may be a viable strategy in reducing cardiovascular disease prevalence at the population level.

## Background

Cardiovascular disease in industrialized countries, including those in the European Union, remains a significant public health problem [1–3]. Each year cardiovascular disease causes over 4 million deaths in Europe and over 1.9 million deaths in the European Union, accounting for 40% of all deaths in the European Union [3]. In France, obesity prevalence significantly increased between 1995 and 2005 for all socio-economic status population sub-groups [4]. Furthermore, in a recent study of French adults, 19.1% of the participants had hypertension [5]. Like elsewhere, in the European Union, cardiovascular disease is costly [6]. For example, the cost of hypertension treatment has increased from 2.6 billion euros in 2000 to 4.4 billion euros in 2006 in France [7].

Neighborhood factors, including neighborhood walkability (including access to walkable destinations such as parks and community design features that can promote walking such as sidewalks), can influence cardiovascular health outcomes across geographies and populations [8–16]. While a large number of neighborhood walkability studies have been conducted, the measures used to examine neighborhood walkability have varied considerably and many of these studies consider only single or few measures of walkable development [17]. Composite measures of neighborhood walkability such as Walk Score (www.walkscore.com) might be useful as predictors of cardiovascular health—as studies that use singe components of neighborhood walkability often generally document fewer relationships and are much less consistent regarding direction of effect [18].

Walk Score is a novel and valid measure of estimating certain aspects of neighborhood walkability, which provides up-to-date geospatial walkability information [18]. However, to date, only a handful of research studies have utilized Walk Score as a measure of neighborhood walkability in connection to cardiovascular health outcomes [18]. Though the association between walkable neighborhoods and multiple cardio-metabolic outcomes has been posited [19], built environment influences on certain cardio-metabolic outcomes such as blood pressure are less frequently examined. Indeed, most such studies focus on physical activity as an outcome, which is further away from diseases and vital health status. The next most commonly analyzed outcome is obesity. It is also important to note that several studies examining the associations of Walk Score on cardiovascular health outcomes have utilized small community-based U.S. samples. For example, in a study of 197 women Supplemental Nutrition Assistance Program (SNAP) participants in eastern North Carolina (U.S.), Walk Score was inversely associated with BMI. [20] Large population-based samples are necessary for detecting meaningful effects as well as for increasing generalizability across geographies and populations. Some recent Walk Score research has used larger population-based samples of adults and found associations between Walk Score and walking (e.g., a study including 4552 adults from the Multi-Ethnic Study of Atherosclerosis [MESA] study in 6 U.S. sites). [21] The current study adds to previous literature by expanding work utilizing Walk Score outside of the U.S. and examining multiple cardiometabolic outcomes. Though some Walk Score studies have been conducted in non-U.S. locations including in Canada [22–25] and Australia [26], this particular study in France will be beneficial in diversifying the literature and among the first such study to be conducted in the European Union. [27, 28] In line with the recent Canadian and Australian research, the current study uses a larger sample size.

The purpose of the current study was to evaluate associations between Walk Score, as a composite measure of walkable neighborhood development, and selected cardiometabolic outcomes, including obesity, hypertension, and heart rate, among adults from the RECORD Study in the Paris metropolitan area. We hypothesized that higher Walk Scores are associated with improved cardiovascular health (e.g., associated with decreased body mass index, blood pressure, and heart rate).

## Methods

### Study Population

The present cross-sectional analysis was based on data from the second wave of the Residential Environment and CORonary heart Disease (RECORD) Cohort (www.record-study.org). [29] Overall, 6003 adult participants were surveyed between January 2011 and March 2014. Among those, 3843 participants had already been enrolled in the RECORD Study in the first wave (2007–2008) and 2160 were new recruits. All participants were recruited as a convenience sample among individuals coming to the IPC Medical Center in Paris for a two-hour medical checkup. As eligibility criteria, participants were 34–84 years old and had to reside at recruitment in one of ten administrative divisions of Paris or in 111 other municipalities selected in the Paris metropolitan area. Among the 6003 participants, 5993 (99.9%) were geocoded based on their residential address in 2011–2014. Additional details on the study have been reported elsewhere. [29] The study protocol was approved by the French Data Protection Authority. The current secondary analysis was determined to be exempt by the New York University School of Medicine Institutional Review Board.

### Measures

#### Body mass index

During the medical checkup, height (using a wall-mounted stadiometer) and weight (using calibrated scales) were recorded by a nurse. [30] Body mass index (BMI) (weight/height^2^ in kg/m^2^) was expressed as a continuous variable and participants were defined as obese (binary variable) if their BMI was >30 kg/m^2^.

#### Waist circumference

Waist circumference (in cm) was measured using an inelastic measurement tape placed midway between the lower ribs and iliac crests on the midaxillary lines and was expressed as a continuous variable.

#### Blood pressure and hypertension

Supine brachial blood pressure was measured by trained nurses three times in the right arm after a 10-minute rest period, using a manual mercury sphygmomanometer. A standard cuff size was used, but a large cuff was employed if necessary. Systolic blood pressure (SBP) and diastolic blood pressure (DBP) were defined as the first and fifth Korotkoff phases, respectively, using the mean of the last two blood pressure measurements. [31] Antihypertensive medication use was assessed as a binary variable with the following questionnaire item: “Are you regularly taking antihypertensive medication?” Participants were defined as hypertensives (binary variable) if their measured SBP was ≥140 mm Hg (mmHg) or if their DBP was ≥90 mmHg or if they had declared antihypertensive medications [32]. Family history of hypertension was self-reported.

#### Resting heart rate

Resting heart rate (RHR) was measured by ECG after a 5 to 7-min rest period [30] and was expressed as a continuous variable. Despite various categorizations in the literature [33–36], RHR was subsequently divided into three categories to be consistent with previous studies [37–40]: <60 beats per minute (bpm), 60–70 bpm, and >70 bpm.

#### Recreational walking

Recreational walking in the residential neighborhood, expressed in minutes per week of walking, was assessed as a continuous variable with the following questionnaire item: “Over the past 7 days, how long in hours and minutes you have walked in total for recreational walks or exercise in your residential neighborhood?”. Absence of walking is coded as 0 min. A binary variable of recreational walking in the residential neighborhood based on the same item distinguished participants with and without recreational walking.

### Individual and neighborhood variables

Gender was coded as a binary variable and age was categorized as follows: 34–49 years old, 50–64 years old, and 65–84 years old. Household status was coded in 3 classes: living alone, as a couple, and as a family. Education was divided into four classes: no education (low), primary and low secondary education (mid-low), high secondary and low tertiary education (mid-high), and upper tertiary education (high). Employment status was coded as employed, unemployed, retired, and other. Self-reported financial strain and nonownership of dwelling were coded with binary variables.

Neighborhood socioeconomic status was defined as the education of the residents in circular buffers of 1 mile of radius centered on the residence of the participants. The educational level of the neighborhood residents was defined as the proportion of residents aged >20 years with an upper tertiary education (2010 Census). This variable was divided into 4 categories (quartiles) comprising a similar number of participants. We selected these covariates based on past research, including our coding scheme and our relevant past work in the RECORD Cohort Study [41–44].

### Risk factors of Cardiometabolic outcomes

Alcohol consumption was coded in four categories: Never drinkers, former drinkers (who declared not drinking alcohol anymore), light drinkers (between one and ≤two glasses per day for women and between one and ≤three glasses per day for men), and drinkers (>two glasses per day for women and >three glasses per day for men). For smoking, we distinguished between nonsmoker, former smoker, and smoker. A 16-item questionnaire that assessed the score of adherence to the traditional Mediterranean diet was derived from previously validated questionnaires [45, 46]. We assigned values of 0 or 1 to each item and added them. The variable of diet score was coded as a continuous variable.

### Neighborhood walk score

Originally developed by Front Seat Management, Walk Score® (available at: www.walkscore.com) calculates neighborhood walkability using a web-based algorithm for a 1-mile radius of an address. The algorithm uses a distance-decay function. If the closest establishment of a certain type is within 0.25 miles, Walk Score assigns the maximum number of points for that type. No points are awarded for destinations more than 1-mile away. It uses publicly available data and places added by the Walk Score user community. Walk Score divides facilities into various categories including: educational (e.g., schools), retail (e.g., bookstores), food (e.g., restaurants), recreational (e.g., gyms), and entertainment (e.g., movie theaters). Each destination type is weighted equally. Walk Scores assigned to the various categories are summed and normalized into a continuous score ranging from 0 to 100. Higher scores indicate increased walkability. It is important to note that Walk Score has been validated against several features of the built environment (e.g., retail destinations, service destinations, parks, street connectivity, and residential density) created using geographical information systems (GIS) technology [47–50]. In addition, Walk Score has been associated with individuals’ perception of their built environment (e.g., perceived physical activity facilities) [47]. For this study, Walk Scores were obtained from Walk Score in April and June 2014 for the geographic coordinates of each participant’s street address in the second wave of the RECORD Study. The traditional Walk Score was used in this analysis, which uses an ‘as the crows flies’ distance, as opposed to the Street Smart Walk Score which accounts for the pedestrian friendliness factors (e.g., average block length), because these were the only measures available to us. To minimize spatial misclassification, [11] we used participants’ actual address, as opposed to an administrative area (e.g., census tract). Walk Scores were examined continuously and using categories designated by Walk Score: 0–24, “Very Car-Dependent” (almost all errands require a car); 25–49, “Car-Dependent” (a few amenities within walking distance); 50–69, “Somewhat Walkable” (some amenities within walking distance); 70–89, “Very Walkable” (most errands can be accomplished on foot); and 90–100, “Walker’s Paradise” (daily errands do not require a car).

### Statistical analysis

We first conducted descriptive analyses, calculating means and standard deviations for study variables. Multilevel linear regression models (with a random effect at the census block group neighborhood level) were then estimated with the restricted maximum likelihood approach to examine associations for Walk Score with the continuous outcomes: BMI, waist circumference, SBP, DBP, RHR, and neighborhood recreational walking (in minutes per week). The multilevel linear regression models were estimated with participants nested within 2084 census block group neighborhoods. These units, defined for the Population Census, were relatively homogeneous in terms of sociodemographic and housing characteristics. Overall, 33% of the census block groups comprised only one participant. This is not a problem with this methodology in which neighborhoods with only one participant do not contribute to the neighborhood-level variance. The distribution of the residuals were approximately normal.

In the analysis of BP, since controlling for antihypertensive medication use in this analysis can introduce bias (as the treatment is itself a consequence of a high underlying BP level) [51], we added a constant to the observed BP (10 mmHg for SBP and 3.24 mmHg for DBP) in treated subjects to account for the treatment effect on BP, instead of adjusting (as previously recommended) [51, 52].

Since the prevalence of obesity and hypertension respectively reached 12.3% and 34.1%, modified Poisson [53] regression models (yielding a robust variance estimator based on the Generalized Estimating Equations) were estimated to examine associations of Walk Score with the binary variables of obesity, hypertension, and neighborhood recreational walking, and also with resting heart rate as a categorical variable, in order to derive relative risks.

For each sample, a null model was first estimated. Then, models for the associations with Walk Score were adjusted for participant-level variables. Models were then adjusted for neighborhood-level education. For the linear models, we report beta coefficients expressing increases/decreases: in kg/m^2^ of BMI, in cm of waist circumference, in mmHg of BP, in bpm of resting heart rate, and in minutes per week of neighborhood recreational walking. These increases/decreases were associated: 1) with a one point increase in the continuous variable of Walk Score; and 2) with “Very / Car-dependent”, “Somewhat Walkable”, “Very Walkable”, in comparison with “Walker’s Paradise” (categorical variable of Walk Score). For the modified Poisson models, we reported prevalence ratios for being obese, for being hypertensive, for a higher resting heart rate, and for practicing recreational walking in one’s residential neighborhood associated with “Very/Car-dependent”, “Somewhat Walkable”, “Very Walkable”, in comparison with “Walker’s Paradise” (categorical variable of Walk Score). The categories “Car-Dependent” and “Very Car-Dependent” were grouped into one category: “Very/Car-Dependent” in order to ensure sufficient statistical power, which has also been done in previous Walk Score research [54]. All analyses were conducted with SAS version 9.3 (Cary, North Carolina, U.S.).

## Results

### Descriptive statistics

Table 1 shows descriptive statistics for Walk Scores. The largest group of participants lived in Walker’s Paradise (48.3%), with a mean Walk Score of 95.7 (SD = ±3.1), followed by Very Walkable neighborhoods (36.4% of participants, mean Walk Score = 80.4, SD = ±5.6). The 10th, 50th, and 90th percentiles of the Walk Score in the sample were 65, 88, and 98 respectively. Table 2 shows descriptive statistics for the RECORD participants. The mean age of the sample was 53.7 years and most participants were male (67.3%). In addition, most participants reported living as a couple, having a high level of education, and being employed. Approximately 60% reported owning their dwelling. The prevalence of recreational walking in the neighborhood was 68.7%.

**Table 1:** Descriptive statistics for Walk Scores

Walk Score	Mean (Standard Deviation)	Range^a^
Overall (*n* = 5993)	84.2 (±15.0)	100
Very/Car-Dependent (*n* = 155)	32.7 (±14.8)	49
Somewhat Walkable (*n* = 764)	62.0 (±5.1)	18
Very Walkable (*n* = 2182)	80.4 (±5.6)	18
Walker’s Paradise (*n* = 2892)	95.7 (±3.1)	10

^a^The range was calculated as the maximum minus the minimum score

**Table 2:** Descriptive statistics for the RECORD participants (*N* = 5993)

Individual socio-demographic characteristics	Participants (*N* = 5993) % (*N*)
Gender	
Male	67.3% (4035)
Female	32.7% (1958)
Age	
34–49	40.1% % (2403)
50–64	41.3% (2474)
65–84	18.7% (1116)
Household status	
Living alone	27.2% % (1136)
Living as a couple	61.7% (2580)
Living as a family	11.1% (463)
Individual education	
High	41.6% (2476)
Mid-high	29.7% (1768)
Mid-low	22.0% (1311)
Low	6.7% (400)
Financial strain	
Yes	14.5% (868)
No	85.5% (5125)
Employment status	
Employed	60.1% (3603)
Unemployed	13.2% (791)
Retired	23.5% (1405)
Other	3.2% (194)
Ownership of dwelling	
Owner	59.8% (3586)
Nonowner	40.2%(2407)

### Association between neighborhood walk score and Cardiometabolic outcomes

The distribution of cardiometabolic outcomes according to the Walk Score is shown in Table 3. Living in more walkable neighborhoods was associated with a lower BMI, a smaller waist circumference, increased recreational walking in the residential neighborhood, a lower SBP, a lower DBP, and a lower RHR (all p for trend <0.01).

**Table 3:** Distribution of cardiometabolic outcomes according to the Walk Score

Walk Score	BMI Mean ± SD (P25; P75)	Waist circumference Mean ± SD (P25; P75)	Recreational walking Mean ± SD (P25; P75)
	*N* = 5971	*N* = 5697	*N* = 5987
Very/Car-Dependent Somewhat Walkable Very Walkable Walker’s Paradise	26.3 ± 3.9 (23.9; 28.1) 25.9 ± 3.9 (23.1; 28.0) 25.9 ± 4.2 (23.1; 28.1) 25.1 ± 3.9 (22.5; 27.3)	90.1 ± 11.2 (84.0; 96.0) 88.0 ± 11.4 (80.0; 96.0) 88.4 ± 11.9 (80.0; 96.0) 86.8 ± 11.7 (79.0; 94.0)	115.4 ± 175.4 (0; 180.0) 99.3 ± 155.6 (0; 120.0) 113.4 ± 181.2 (0; 120.0) 132.4 ± 182.6 (0; 180.0)
P For Trend^a^	*<.0001* ^a^	*<.0001* ^a^	*<.0001* ^a^
Walk Score	SBP Mean ± SD (P25; P75)	DBP Mean ± SD (P25; P75)	RHR Mean ± SD (P25; P75)
	*N* = 5883	N = 5883	*N* = 5922
Very/Car-Dependent Somewhat Walkable Very Walkable Walker’s Paradise	133.0 ± 16.2 (123.0; 143.0) 132.8 ± 16.8 (121.0; 142.0) 132.7 ± 16.2 (121.5; 141.5) 131.5 ± 16.5 (120.0; 141.5)	80.0 ± 9.6 (73.0; 86.5) 79.2 ± 9.4 (73.0; 85.0) 78.9 ± 9.5 (72.5; 85.0) 77.9 ± 9.2 (71.5; 83.5)	61.0 ± 9.1 (55.0; 67.0) 62.1 ± 9.6 (55.0; 68.0) 61.8 ± 10.0 (55.0; 68.0) 60.9 ± 9.8 (54.0; 67.0)
P For Trend^a^	*0.0005* ^a^	*<.0001* ^*a*^	*0.0004* ^*a*^

^a^P values for trends were estimated from the Jonckheere-Terpstra test

*Abbreviations*: *BMI* Body Mass Index, Recreational walking in the residential neighborhood, *SBP* Systolic Blood Pressure, *DBP* Diastolic Blood Pressure, *RHR* Resting Heart Rate

These relationships persisted in adjusted models (Table 4). After adjustment for individual variables, neighborhood variables, and risk factors for CVD outcomes, we found that Walk Score was associated with decreased BMI (β: -0.010, 95% CI: -0.019 to −0.002 for a unit increase in Walk Score), decreased waist circumference (β: -0.031, 95% CI: -0.054 to −0.008), increased neighborhood recreational walking (β: +0.73, 95% CI: +0.37 to +1.10), decreased SBP (β: -0.030, 95% CI: -0.063 to +0.004), decreased DBP (β: -0.028, 95% CI: -0.047 to −0.008), and decreased RHR (β: -0.026, 95% CI: -0.046 to −0.005).

**Table 4:** Associations estimated between continuous Walk Score and cardiometabolic outcomes, adjusted for individual variables, neighborhood variables and risk factors related to cardiometabolic outcomes

Cardiometabolic outcomes	*N*	β 95% CI	ICC Null.	ICC Adj.
Body Mass Index	5971	-0.010 -0.019 to −0.002	0.042	0.027
Waist circumference	5697	-0.031 -0.054 to −0.008	0.031	0.018
Systolic blood pressure	5883	-0.030 -0.063 to +0.004	0.016	0.009
Diastolic blood pressure	5883	-0.028 -0.047 to −0.008	0.018	0.011
Resting heart rate	5922	-0.026 -0.046 to −0.005	-^b^	-^b^
Recreational walking^a^	5987	+0.73 + 0.37 to +1.10	0.008	-^b^

*Abbreviations*: *ICC Null* Intra-class Correlation Coefficient for the null model (only age and gender), *ICC Adj* Intra-class Correlation Coefficient, after adjustment for Walk Score and individual, neighborhood factors, and risk factors of the cardiometabolic outcomes (except recreational walking). Walk score was expressed as a continuous score ranging from 0 to 100 in the residential neighborhood. The variables of BMI (in kg/m^2^), waist circumference (in centimeter), recreational walking (in minutes/week of walking), and resting heart rate (in beats/min) were expressed as continuous variables. SBP and DBP (in mmHg) were expressed as continuous variables with 10 mmHg added to the observed SBP values and with 3.24 mmHg added to the observed DBP values for the participants on antihypertensive treatment. Multilevel linear regression models were estimated after excluding individuals with missing values for Walk Score, for census block group neighborhoods, and for each cardiometabolic outcome

^a^Multilevel linear regression models were estimated after adjusting for individual and neighborhood variables ^b^In certain models, the between-neighborhood variance could not be estimated after adjustment, possibly due to the very low level of clustering

Compared to living in Walker’s Paradise, living in very walkable and particularly in very/car dependent neighborhoods was associated with a higher BMI, increased obesity prevalence, and a larger waist circumference (Table 5). For example, individuals living in a very/car-dependent neighborhood were 1.66 (95% CI: 1.09 to 2.52) times more likely to be obese. Living in very/car dependent neighborhoods, compared to living in Walker’s Paradise, was associated with higher DBP (Table 5). More specifically, individuals living in a very/ car-dependent neighborhood had a 1.84 (95% CI: 0.21 to 3.46) mmHg higher DBP. Walk Score categories were not significantly associated with SBP or RHR. Lastly, living in less walkable neighborhoods, compared to living in Walker’s Paradise, was associated with decreased neighborhood recreational walking (Table 5). For example, individuals living in a very/car-dependent neighborhood had approximately 45 (95% CI, −75.91 to −14.01) fewer minutes of neighborhood recreational walking over the previous 7 days.

**Table 5:** Associations estimated between categorical Walk Score and cardiometabolic outcomes, adjusted for individual variables, neighborhood variables and risk factors related to cardiometabolic outcomes

Cardiometabolic outcomes	Walker’s Paradise	Very Walkable	Somewhat Walkable	Very/Car-Dependent
Continuous outcomes		β 95% CI	β 95% CI	β 95% CI
Body Mass Index	Ref	+0.30 + 0.04 to +0.55	+0.11 -0.26 to +0.48	+0.88 + 0.18 to +1.59
Wait circumference	Ref	+0.75 + 0.06 to +1.45	+0.44 -0.55 to +1.42	+2.75 + 0.86 to +4.63
Systolic blood pressure	Ref	+0.47 -0.53 to +1.48	+0.77 -0.68 to +2.21	+1.62 -1.18 to +4.43
Diastolic Blood pressure	Ref	+0.39 -0.20 to +0.97	+0.61 -0.22 to +1.45	+1.84 + 0.21 to +3.46
Resting heart rate	Ref	+0.58 -0.04 to +1.20	+0.85 -0.04 to +1.74	+0.68 -1.07 to +2.42
Recreational walking^a^	Ref	−14.46 -25.51 to −3.42	−26.96 -42.84 to −11.08	−44.96 -75.91 to −14.01
Categorical outcomes		PR 95% CI	PR 95% CI	PR 95% CI
Obese	Ref	1.23 1.04 to 1.46	1.02 0.80 to 1.30	1.66 1.09 to 2.52
Hypertension	Ref	0.99 0.91 to 1.07	1.00 0.89 to 1.12	1.10 0.87 to 1.39
Resting heart rate	Ref	1.02 0.99 to 1.04	1.02 0.98 to 1.07	1.04 0.96 to 1.12
Recreational walking^a^	Ref	0.96 0.92 to 1.01	0.93 0.87 to 0.99	0.93 0.82 to 1.06

Multilevel linear regression and modified Poisson regression models were estimated after excluding individuals with missing values for Walk Score, for census block group neighborhoods, and for each cardiometabolic outcome. These models estimated associations between categorical Walk Score and continuous and categorical cardiometabolic outcomes, adjusted for individual, neighborhood factors, and risk factors of these cardiometabolic outcomes, such as alcohol consumption, smoking habits, and the quantitative score of adherence to Mediterranean diet

^a^These models were estimated after adjusting for individual and neighborhood variables

## Discussion

The current study builds on and addresses certain limitations of existing built environment research, by using Walk Score data, in a unique population-based sample of adults in the Paris metropolitan area (Paris, France). This is one of the largest studies to examine relationships between Walk Score and selected cardiometabolic outcomes. Additionally, this is one of few Walk Score studies to be conducted outside of the U.S., and one of the first in Europe to our best knowledge. In this study, we found that living in more walkable neighborhoods was associated with a lower BMI, a smaller waist circumference, increased recreational walking in the residential neighborhood, a lower SBP, a lower DBP, and lower resting heart rate. The reported relationships were modest, which is likely due to the large causal distance between the environmental Walk Score and the cardiometabolic outcomes of interest. Despite their limited clinical significance for a given individual, these findings may have a public health importance as they contribute with other factors (e.g., socioeconomic status) to shape population-level disparities in health. Our results were robust to different ways to categorize Walk Score and robust to covariate adjustment. We note that reductions in diastolic BP are not nearly as important as reductions in systolic blood pressure, which is the principal driver of long term cardiovascular risk.

Previous studies, frequently using smaller samples, have examined relationships between Walk Score and cardiovascular health outcomes in the U.S. context predominantly, and our results are comparable to these studies, including those across geographies. For instance, previous research has found associations between Walk Score and BMI [20, 24, 55, 56], hypertension [22], heart rate [55] and physical activity or walking [21, 23–26, 54–57]. The vast majority of these studies analyze associations between Walk Score and physical activity or walking and often analyze transportation walking. Unfortunately, the second wave of the RECORD Study did not have transportation walking and in a previous analysis with the first wave of the RECORD Study (as opposed to the second wave in the present study) found densities of destinations (Walk Score was not available) to be associated with recreational walking [41]. We concluded that this typical predictor of transportation walking was also relevant to recreational walking. Recreational walking could be associated with Walk Score because one could choose to walk in a neighborhood the look at different amenities. In a similar study, we sought to explore associations that might emerge between Walk Score, transportation mode choice, and walking at the trip level among Paris adults who were tracked with GPS receivers and accelerometers from the RECORD GPS Study (*n* = 227 participants; 6969 trips) [58]. When we adjusted trip-level associations between Walk Score and walking only in the trip, the findings indicated that there was an association between walkable neighborhood at the trip origin and the trip destination and an increased odds of walking in the trip—assessed through the survey. Furthermore, we observed the number of steps per 10 min (which was assessed with accelerometry) to be cumulatively higher for trips that originated and ended in walkable neighborhoods (i.e. “Very Walkable”). In addition, similar to our study findings, Hisch and her colleagues found that Walk Score was associated with leisure walking [21].

There are several potential explanations for our findings. Walk Score primarily measures access to destinations and as such people may be walking to these proximate destinations, increasing their physical activity and reducing clinical cardiovascular disease-related outcomes. Moreover, it is possible that increases in neighborhood destinations may be related to pleasantness of the neighborhood, which could also be associated with improved overall cardiovascular health through pathways other than strictly walking to these destinations (such as recreational, non-purposive walking).

Although the results from the current study need to be confirmed with strong study designs such as a prospective cohort study design, findings from this study suggest that changes to the built environment can be implemented. Perhaps, providing nearby access to a wide range of destinations may have its importance for cardiovascular disease prevention. Pursuing these built environment interventions may require support from multiple sectors, including urban planners and economic developers [17].

### Future research directions

Walk Score and other composite measures of neighborhood walkability (e.g., Walk Shed) can be used in future research to elucidate their potential connections to health outcomes such as cardio-metabolic outcomes examined in the present study including obesity and hypertension. When possible, these future investigations should utilize a prospective cohort research design to improve causal inference. We note that emerging longitudinal studies of Walk Score and health outcomes have been conducted. [56] Historical Walk Scores (currently not available) would be useful to integrate into existing population health datasets to conduct longitudinal analyses. Furthermore, studies should be conducted across the European Union and other global geographic locations with various samples (e.g., young children and older adults). Studies using standardized analytical designs should be conducted to compare Walk Score effects, e.g., between Europe and North America. Such cross-national studies can potentially demonstrate the salience of neighborhood walkability on cardiovascular health. Finally, in addition to the traditional Walk Score, future studies can examine the cardiovascular health effects of the Street Smart Walk Score (an enhanced version of Walk Score that uses walking distances rather than crow-flies distances to calculate the score) as well as Walk Score’s Transit Score (a measure of how well a location is served by public transit) and Walk Score’s new Bike Score (a measure of whether a location is good for biking).

### Study strengths and limitations

This study has a number of strengths including the large and diverse population-based sample encompassing a variety of environmental conditions in a European metropolitan area and several objectively measured cardio-metabolic outcomes. However, there are a number of limitations that also should be recognized. First, we conducted a cross-sectional analysis. Therefore, while we found an association between neighborhood Walk Score and cardiovascular-related factors, these relationships are not causal. Moreover, we only considered selected cardiometabolic outcomes, and others would have been relevant to analyze, e.g., inflammatory markers. We did not adjust for whether specific participants with particularly healthy cardiovascular profiles chose to live in highly walkable neighborhoods. However, as previously discussed, a recent prospective cohort study reported relationships between Walk Score and cardiovascular health outcomes (i.e. walking and BMI) [56] and recent studies suggest that the impact of neighborhood self-selection is minimal in studies of neighborhood built environments and cardiovascular health [59]. In addition, while Walk Score has been validated in the U.S., no Walk Score validation research has been conducted in Paris in France more broadly or in Europe [18]. We have no reason however, to believe that our Walk Score data for the Paris metropolitan area is substantially different than data in the U.S.-based studies, especially because most validation studies have been in urban settings. Previous Walk Score validation research, for example, has found higher correlations between Walk Scores and population density in geographic locations with increasing population density [50] and Paris is a densely populated city. This study used the standard Walk Score, which may be a limitation. As described previously, the standard Walk Score does not take into account for pedestrian friendliness factors intersection density. The Street Smart Walk Score was not available to us for the Paris metropolitan area at the time of data collection. However, previous research has found strong correlations between the standard Walk Score and the Street Smart Walk Score [21]. We recognize that Walk Score is a useful proxy for only certain neighborhood walkability indicators (e.g., retail destinations and intersection density) [47, 50]. Walk Score does not consider certain neighborhood-related characteristics (e.g., traffic) and weights all destinations equally, which may result in misclassification compared to a “true” walkability indicator. In addition, spatial polygamy [60–63] and the uncertain geographic context problem [64, 65] may be concerns that we did not address in this study. For this study, we only had Walk Scores for participants’ residential addresses, and for example not for their work locations [66, 67]. Generalizability may also be a concern. For example, findings from this study might not be generalizable to urbanized non-industrialized countries. Finally, residual confounding might be a concern due to other not mentioned reasons. For example, we did not control for individual-level race/ethnicity in the current study. In our previous U.S.-based research, we control for race/ethnicity as a covariate. Race/ethnicity is not typically considered in French studies, however, for ethical reasons. Thus this variable was not available for us to analyze.

## Conclusion

Walk Score is a valid measure for estimating certain aspects of neighborhood walkability and provides up-to-date geospatial walkability information [18]. In a population-based sample of adults in the Paris metropolitan area, we found that living in a highly walkable neighborhood was associated with improved cardiovascular health (e.g., decreases in BMI and blood pressure). Designing walkable neighborhoods may be a viable strategy in reducing cardiovascular disease prevalence at the population level.

## Abbreviations

BMI: Body mass index
DBP: Diastolic blood pressure
ICC Adj.: Intra-class Correlation Coefficient, after adjustment for Walk Score and individual, neighborhood factors, and risk factors of the cardiometabolic outcomes (except recreational walking).
ICC Null: Intra-class Correlation Coefficient for the null model (only age and gender)
RECORD: Residential Environment and CORonary heart Disease
RHR: Resting heart rate
SBP: Systolic blood pressure
SNAP: Supplemental Nutrition Assistance Program
